# *Myanmarorchestia victoria* sp. nov. (Crustacea, Amphipoda, Talitridae), a new species of landhopper from the high altitude forests in Myanmar

**DOI:** 10.24272/j.issn.2095-8137.2017.067

**Published:** 2017-09-18

**Authors:** Ya-Mi Zheng, Zhong-E Hou

**Affiliations:** ^1^Key Laboratory of Zoological Systematics and Evolution, Institute of Zoology, Chinese Academy of Sciences, Beijing 100101, China; ^2^Southeast Asia Biodiversity Research Institute, Chinese Academy of Sciences, Yezin Nay Pyi Taw 05282, Myanmar

**Keywords:** Taxonomy, Mt. Victoria, *COI* gene, Leaf litter, Morphology, New species

## Abstract

*Myanmarorchestia victoria* **sp. nov**. is described from high altitude habitats in Myanmar. The new species differs morphologically from its congeners by palp of maxilliped narrow; sexually dimorphic gnathopod Ⅱ, propodus of male chelate and propodus of female mitten-shaped; and dimorphic uropod Ⅱ, outer ramus of male with small teeth distally, outer ramus of female with three distal spines. Analysis of DNA barcode sequences and niche distinctiveness support recognition of the new species.

## INTRODUCTION

The landhopper genus *Myanmarorchestia* Hou & Zhao (2017) currently includes two species, distributed in high altitude forests of Mt. Victoria, Myanmar. *Myanmarorchestia* species can be found in 3 000 m a.s.l. or higher, and show some vertical distribution patterns. For example, *Myanmarorchestia* *peterjaegeri* Hou & Zhao (2017) occurs above 2 000 m a.s.l., while *M. seabri* Hou & Zhao (2017) inhabits understorey leaf litter around 1 500 m a.s.l.. The genus *Myanmarorchestia* has the characteristic chelate, sexually dimorphic gnathopod Ⅱ, simplidactylate pereopods and complexly lobed gills to adapt to terrestrial environments.

Mt. Victoria (Nat Ma Taung National Park) is situated between the Indian subcontinent and Asian continent, and harbours endemic montane species (Jäger, 2015; Jäger & Minn, 2015). From November 2016–April 2017, five field trips were organized by the Southeast Asia Biodiversity Research Institute (SABRI), Chinese Academy of Sciences (CAS), to explore the biodiversity of Myanmar. Following a detailed examination of the specimens, three *Myanmarorchestia* species were discovered from Mt. Victoria. Of the three *Myanmarorchestia* species, two species have been published (Hou & Zhao, 2017). In the current study, the third one, *Myanmarorchestia victoria* **sp. nov.**, is described and illustrated. Moreover, DNA barcodes of the new species are obtained to confirm its distinctiveness.

## MATERIALS AND METHODS

### Sampling

**Figure 1:**
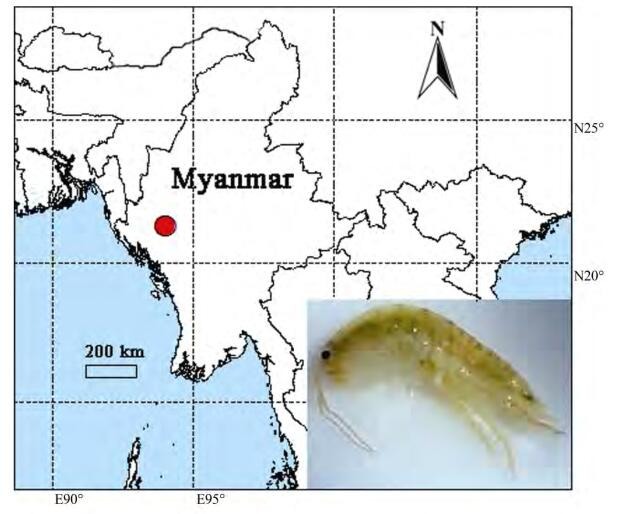
Collection locality of *Myanmarorchestia victoria* sp. nov. from Myanmar (red circle=type locality) (photo by Jiang-Lang Wu)

Fieldworks were conducted in Mt. Victoria, Chin State, Myanmar (Figure 1) from November–December 2016 and from April–May 2017. The specimens were collected by sieving forest floor litter. Samples were preserved in 95% ethanol in the field, then deposited at –20 ℃ refrigerator for long preservation. Type specimens are lodged in the Institute of Zoology, Chinese Academy of Sciences (IZCAS), Beijing.

### Morphology observation

The body length was recorded by holding the specimen straight and measuring the distance along the dorsal side of the body from the base of the first antenna to the base of the telson. All dissected appendages were mounted on slides according to the methods described by Holsinger (1967), and were drawn using a Leica DM2500 compound microscope equipped with a drawing tube. Terminology and taxonomic descriptions follow Morino (2014). The holotype specimen was used for morphological observation, while one paratype specimen was used for both morphological and molecular parts.

### DNA sequencing and *COI* genetic distance calculation

DNA barcode of the mitochondrial cytochrome oxidase subunite I (*COI*) was amplified and sequenced to obtain the genetic distances between morphologically similar species and confirm identifications (Hou *et al*., 2009; Suzuki *et al*., 2017). The primers used are CRUSTF2 (5′-GGTTCTTCTCCACC AACCACAARGAYATHGG-3′) and HCO2198 (5′-TAAACTT CAGGGTGA CCAAAAAATCA-3′). Genomic DNA extraction, amplification and sequencing procedures were performed as in Hou *et al*. (2007). The new sequence was deposited in GenBank.

The *COI* gene sequences were manually aligned, because no indels were observed. Genetic uncorrected *p*-distances among the known *Myanmarorchestia* taxa were calculated using MEGA7.0.16 (Kumar *et al*., 2016).

## TAXONOMY

**Family Talitridae Rafinesque (1815)**

**Genus *Myanmarorchestia*Hou & Zhao (2017)**

**Figure 2:**
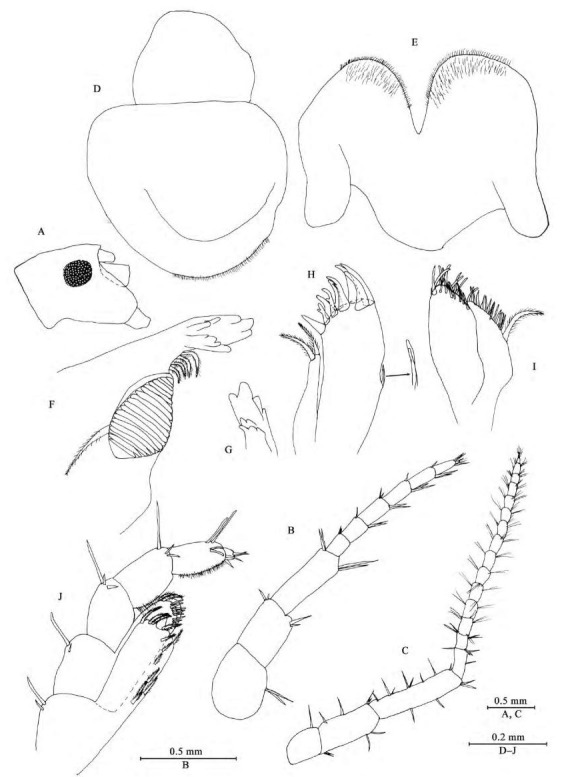
*Myanmarorchestia* *victoria* sp. nov., male holotype (IZCAS-I-A2087-1)

**Figure 3:**
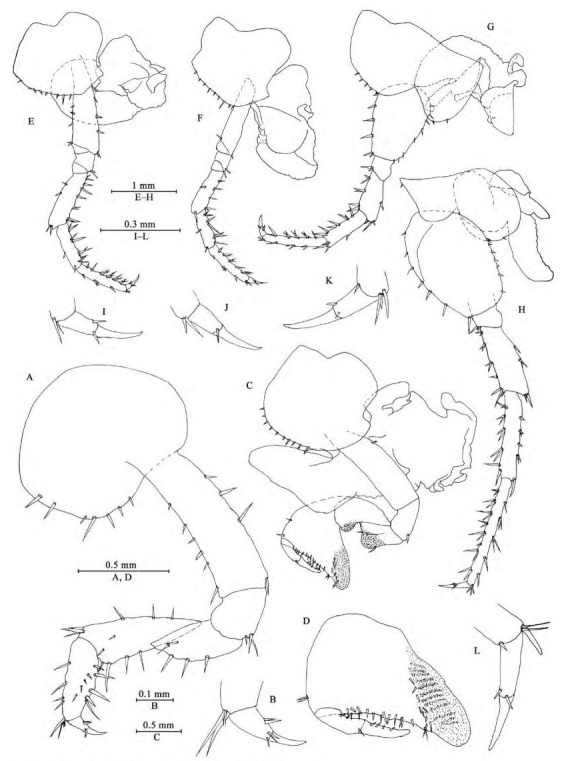
*Myanmarorchestia* *victoria* sp. nov., male holotype (IZCAS-I-A2087-1)

**Figure 4:**
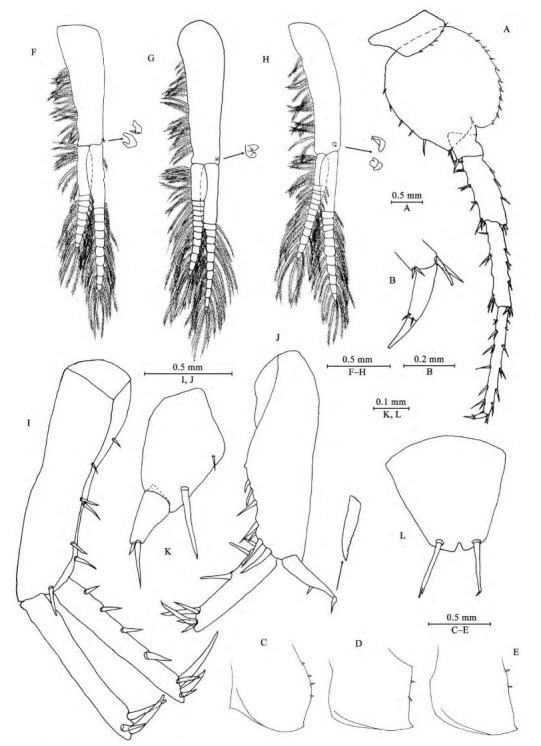
*Myanmarorchestia* *victoria* sp. nov., male holotype (IZCAS-I-A2087-1)

**Figure 5:**
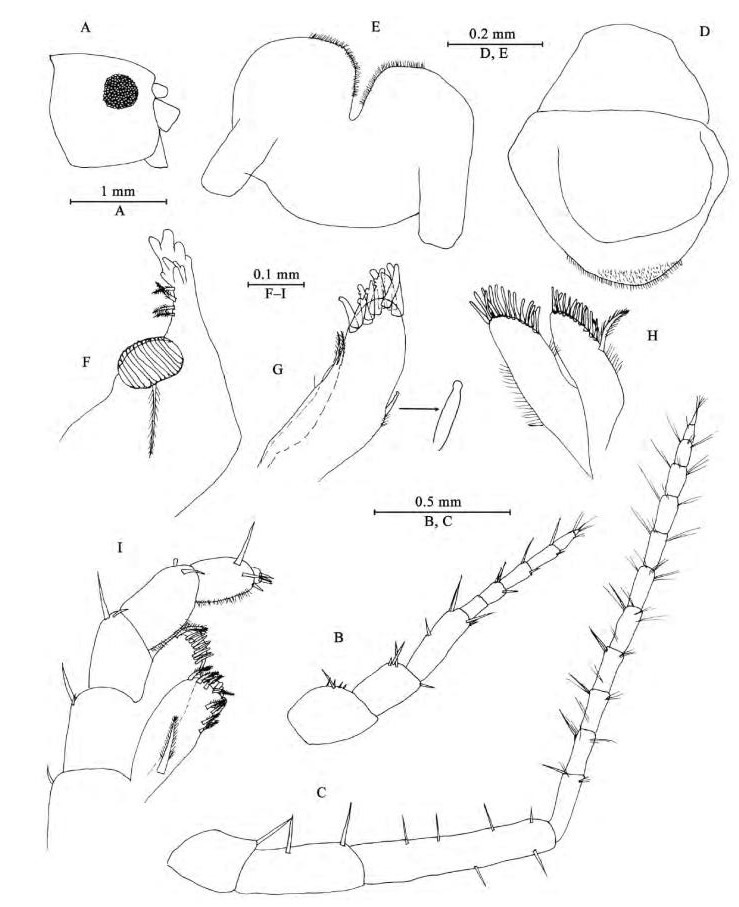
*Myanmarorchestia* *victoria* sp. nov., female paratype (IZCAS-I-A2087-2)

**Figure 6:**
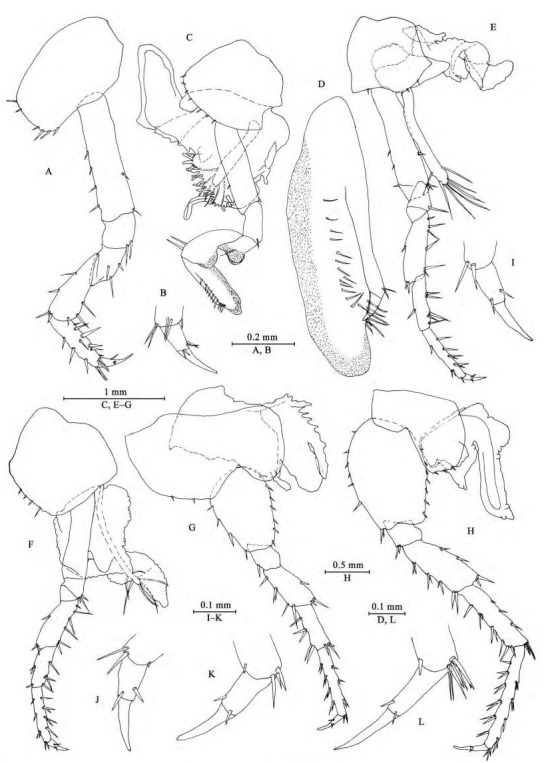
*Myanmarorchestia* *victoria* sp. nov., female paratype (IZCAS-I-A2087-2)

**Figure 7:**
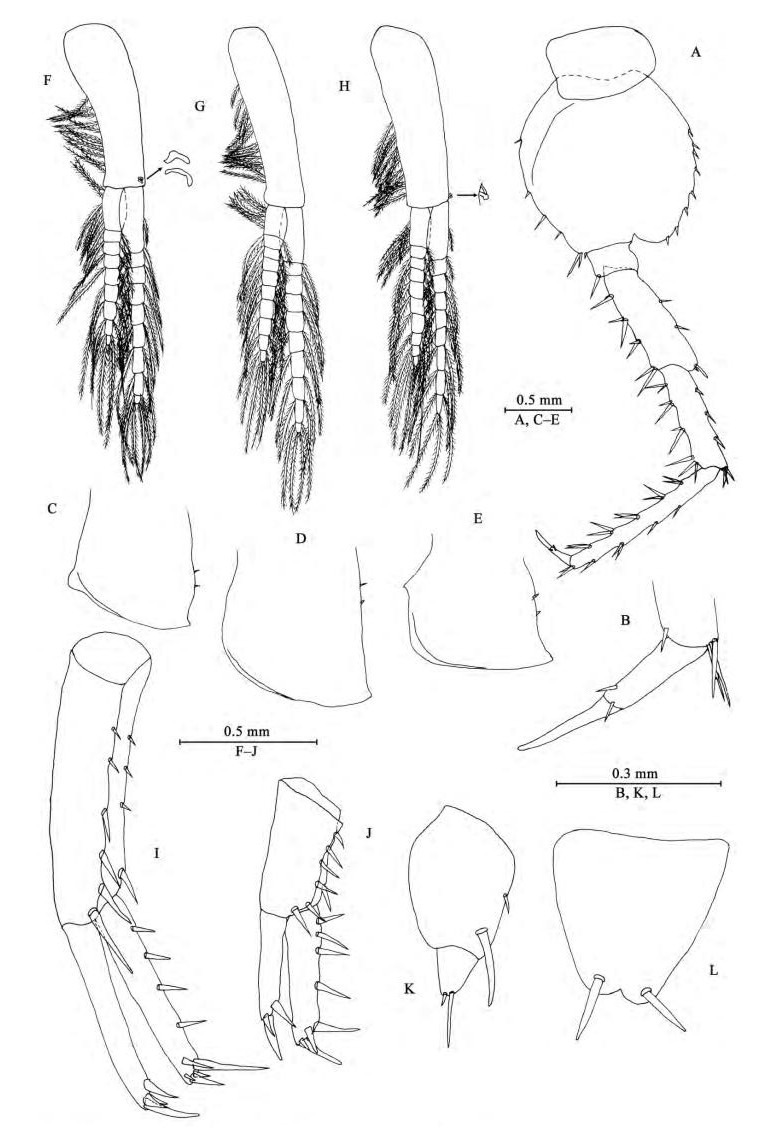
*Myanmarorchestia* *victoria* sp. nov., female paratype (IZCAS-I-A2087-2)

***Myanmarorchestia victoria* Hou sp. nov. (Figures 1–7)**

**Material examined:** Holotype: male (IZCAS-I-A2087-1), 14 mm, near 17.5 km of the roadside between Kanpetlet to Nat Ma Taung National Park, Chin State, Myanmar (E93.94°, N21.22°), altitude 2 654 m a.s.l., collected by J. Wu and Z. Chen on April 30, 2017. Paratype: female (IZCAS-I-A2087-2), 11 mm, same data as holotype, GenBank accession No. MF969263; paratypes, two males and one female (IZCAS-I-A2087-3).

**Etymology:** The species name is derived from the type locality, noun in apposition.

**Diagnosis:** The new species assigns to *Myanmarorchestia* on the basis of the following morphological characteristics: (1) simple gnathopod Ⅰ in both sexes; (2) sexually dimorphic gnathopod Ⅱ, propodus of male chelate and produced on ventral margin, propodus of female mitten-shaped; (3) simplidactylate pereopods Ⅲ–Ⅶ; and (4) complexly lobed coxal gills. The new species is characterized by a combination of the following morphological characteristics: (1) mandible spine row with four plumose setae; (2) maxilliped palp article 2 narrow, article 3 not lobate, article 4 distinct; (3) gnathopod Ⅰ coxal plate not produced on anterior margin; (4) gnathopod Ⅱ strongly dimorphic, propodus of male produced, triangle-shaped, propodus of female mitten-shaped; (5) uropod Ⅱ sexually dimorphic, outer ramus of male weak, with small teeth distally; and (6) uropod Ⅲ peduncle with one strong posterodistal spine.

**Description of holotype male (IZCAS-I-A2087-1), 14 mm**

**Head:** eyes rounded, medium in size, about 35% of head length (Figure 2A).

*Antenna Ⅰ* (Figure 2B): reaching 36% of antenna Ⅱ, peduncle articles 1–3 in length ratio 1.0: 0.8: 1.2; flagellum with seven articles (six large ones and one tiny distal one), a little shorter than peduncle, each article with short distal setae.

*Antenna Ⅱ* (Figure 2C): peduncle articles 3–5 in length ratio 1.0: 1.7: 2.5, with setae on anterior and posterior margins; flagellum with 15 articles, each article with setae on dorsal and ventral margins.

*Upper lip* (Figure 2D): ventral margin rounded, bearing minute setae.

*Mandible* (Figures 2F, G): incisor of left mandible with five teeth; lacinia mobilis with four teeth; spine row with four plumose setae; molar with a plumose seta; incisor of right mandible with four teeth, lacinia mobilis bifurcate, with small teeth.

*Lower lip* (Figure 2E): inner lobes indistinct, outer lobes covered with thin setae.

*Maxilla Ⅰ* (Figure 2H): inner plate with two terminal strong setae, outer plate with nine apical spines (three of them bifid), palp with one article.

*Maxilla Ⅱ* (Figure 2I): inner plate narrower and shorter than outer plate, with one plumose seta and numerous simple setae on medial margin, outer plate with two rows of apical spines.

*Maxilliped* (Figure 2J): inner plate, with one stout apical spine and 12 plumose setae; outer plate bearing eight simple setae and two plumose setae apically; palp with four articles, articles 1–2 not broad; articles 1–3 subequal in length ratio; articles 2–3 with fine setae; article 3 with two spines on interior margin, two setae on exterior margin and two setae on ventral surface; article 4 small but distinct, with two simple setae apically.

**Pereon**

*Gnathopod Ⅰ* (Figures 3A, B): coxal plate broad, bearing seven setae on ventral margin; basis with short setae on anterior and posterior margins; merus, carpus, and propodus in length ratio 1.0: 1.5: 1.1; merus bearing setae on posterior margin; carpus with setae on anterior and posterior margins; propodus simple, with setae on anterior margin and five spines accompanied by setae on posterior margin; dactylus with two spines on posterior margin and three setae at hinge of unguis.

*Gnathopod Ⅱ* (Figures 3C, D): coxal plate ventral margin with ten setae, posterior process prominent; basis with a fine seta on posterior margin; merus protuberant on posterior margin; carpus 1.7 times as long as wide, with tumescent hump at posterodistal corner; propodus with tumescence, subtriangular, with setae on surface, palm margin anteriorly slant, forming chela, with two rows of spines (a lateral and a medial one); dactylus as long as palm, with setae on posterior margin.

*Pereopod Ⅲ* (Figures 3E, I): coxal plate with posterior cusp, bearing eight setae on ventral margin; basis longest, with spines on anterior and posterior margins; merus, carpus, and propodus in length ratio 1.0: 0.8: 1.0; carpus and propodus with spines on posterior margins; dactylus with two spines at hinge of unguis. Pereopods Ⅲ–Ⅶ simplidactylate.

*Pereopod Ⅳ* (Figures 3F, J): similar but shorter than pereopod Ⅲ; coxal plate with posterior cusp, bearing nine setae on ventral margin; merus, carpus, and propodus in length ratio 1.0: 0.8: 1.1, dactylus weakly pinched.

*Pereopod Ⅴ* (Figures 3G, K): coxal plate bilobed, anterior lobe larger than posterior lobe, bearing five setae and two setae on anterior and posterior lobes, respectively; basis suboval, with four spines on anterior margin and eight setae on posterior margin, anterodistal corner with two spines; merus, carpus, and propodus in length ratio 1.0: 1.1: 1.5, with spines on both margins; dactylus with two spines at hinge of unguis.

*Pereopod Ⅵ* (Figures 3H, L): coxal plate bilobed, anterior lobe much smaller than posterior lobe, bearing one seta on anterior lobe and two setae on posterior lobe; basis suboval, with six spines on anterior margin and seven setae on posterior margin, anterodistal corner with two spines; merus, carpus, and propodus in length ratio 1.0: 1.3: 1.7, with spines on both margins; propodus and dactylus slender, dactylus with two spines at hinge of unguis.

*Pereopod Ⅶ* (Figures 4A, B): coxal plate unilobate, shallow, with five setae on posterodistal margin; basis oval, with five setae on anterior margin and 12 setae on posterior margin, anterodistal corner with two spines; merus, carpus, and propodus in length ratio 1.0: 1.3: 1.6, with spines on both margins; propodus and dactylus slender, dactylus with two spines at hinge of unguis.

*Coxal gills* (Figures 3C, E–H): present on gnathopod Ⅱ and pereopods Ⅲ–Ⅵ, complexly lobed and convoluted; gill of gnathopod Ⅱ broad, with ridged margin; gills of pereopods Ⅲ–Ⅵ sac-shaped.

**Pleon**

*Epimeral plates* (Figures 4C–E): acuminate posterodistally, distal margins without armature; plate Ⅰ with four fine setae on posterior margin; plate Ⅱ with two fine setae on posterior margin; plate Ⅲ with two fine setae on posterior margin.

*Pleopods Ⅰ-Ⅲ* (Figures 4F–H): similar, peduncle with two retinacula on interior margin, exterior margin with dense plumose setae; outer ramus about 85% of peduncle, outer ramus about 70% of inner ramus, both inner and outer rami fringed with plumose setae.

**Urosome**

*Uropods Ⅰ-Ⅲ* (Figures 4I–K): uropod I peduncle longer than rami, with three spines on interior margin and three spines on exterior margin, distolateral spine longer than subdistal one; inner ramus with four spines on interior side and four terminal spines; outer ramus marginally bare, with three terminal spines. Uropod Ⅱ short, peduncle bearing one spine on interior margin and six spines on exterior margin; inner ramus with three spines on interior side and five terminal spines; outer ramus weak, shorter than inner ramus, with one spine on interior side and some small teeth distally (we have examined all three males to confirm this unique state). Uropod Ⅲ peduncle expanded, with one seta on dorsal margin and one strong posterodistal spine; ramus short, about 0.5 times as long as peduncle, with one long slender spine and one short spine apically.

*Telson* (Figure 4L): apically notched, about 7% of depth; each lobe with one apical spine.

**Description of paratype female (IZCAS-I-A2087-2), 11 mm**

**Head (Figures 5A–I):** similar to that of male except *Antenna Ⅱ* peduncle articles 3–5 in length ratio 1.0: 2.0: 3.2; *Maxilliped* inner plate with three or four apical spines.

**Pereon**

*Gnathopod Ⅰ* (Figures 6A, B): propodus with interlocking setae for dactylus.

*Gnathopod Ⅱ* (Figures 6C, D): coxal plate ventral margin with seven setae; basis slender; merus protuberant on posterior margin; carpus with tumescent hump at posterodistal comer, with two setae on anterior margin; propodus mitten-shaped, with tumescence, with setae on surface and palm margin; dactylus shorter than palm margin.

*Pereopods Ⅲ-Ⅶ* (Figures 6E–L, 7A, B): similar to those of male.

*Coxal gills* (Figures 6C, E–H): present on gnathopod Ⅱ and pereopods Ⅲ–Ⅵ, complexly lobed and convoluted; gill of gnathopod Ⅱ broad, with marginal filamentous projections; gill of pereopod Ⅲ and Ⅳ similar, lobed and convoluted, with weakly ridged margins; gill of pereopod Ⅴ with ridged margin; gill of pereopod Ⅵ smallest.

*Oostegites* (Figures 6C, E, F): present on gnathopod Ⅱ and pereopods Ⅲ–Ⅳ, slender, with setae on apical margins; oostegite of pereopod Ⅴ missing.

**Pleon**

*Epimeral plates* (Figures 7C–E): acuminate posterodistally, ventral margins without armature; posterior margins with two fine setae.

*Pleopods Ⅰ–Ⅲ* (Figures 7F–H): similar, peduncle with two retinacula on interior margin, exterior margin with dense plumose setae; outer ramus about 86% of peduncle, outer ramus about 76% of inner ramus, both inner and outer rami fringed with plumose setae.

**Urosome**

*Uropods Ⅰ–Ⅲ* (Figures 7I–K): uropod I (Figure 7I) peduncle longer than rami, with four spines on interior margin and five spines on exterior margin, distolateral spine distinct, longer than subdistal one; inner ramus with four spines on interior side and five terminal spines; outer ramus marginally bare, with four terminal spines. Uropod Ⅱ (Figure 7J) short, peduncle bearing one spine on interior margin and six spines on exterior margin; inner ramus with four spines on interior side and five terminal spines; outer ramus shorter than inner ramus, with three terminal spines. Uropod Ⅲ (Figure 7K) peduncle expanded, with one simple spine on dorsal margin and one strong spine on posterodistal corner; ramus short, about 0.3 times as long as peduncle, with one long slender spine and one short spine apically.

*Telson* (Figure 7L): apically notched, about 5% of depth; each lobe with one apical spine.

**Habitat:** This species was collected from a disturbed primary forest, with bamboo and understorey leaf litter, with altitude 2 654 m a.s.l. in Mt. Victoria.

**Remarks:** *Myanmarorchestia victoria* **sp. nov.** is most similar to *M. seabri* in maxilla I palp with one article, coxal gills convoluted, uropod Ⅱ sexually dimorphic, and telson bare on surface. The new species can be distinguished from *M. seabri* by the following characters (*M. seabri* in parentheses): (1) maxilliped palp article 2 narrow (broad); (2) gnathopod Ⅰ coxal plate not produced on anterior margin (produced proximally); (3) coxal gills of pereopods Ⅳ–Ⅴlobed and convoluted, with no filamentous projections (with ridged margins and filamentous projections); and (4) uropod Ⅲ peduncle with one strong posterodistal spine (two posterodistal spines).

*Myanmarorchestia victoria* **sp. nov.** can be distinguished from *M. peterjaegeri* by the following characters (*M. peterjaegeri* in parentheses): (1) palp of maxilla Ⅰ with one article (with two small articles); (2) maxilliped palp article 2 narrow (broad); (3) coxal gills lobed and convoluted, with no filamentous projections (with more filamentous projections); and (4) uropod Ⅱ sexually dimorphic, outer ramus of male weak, with small teeth distally (similar for male and female, with three or four terminal spines). Distinguishing features of *Myanmarorchestia* species can be found in the key below.

The uncorrected *p*-distance among the three *Myanmarorchestia* species ranged from 14.8%–18.8% for *COI* gene. The new species differed from *M. peterjaegeri* and *M. seabri* by 17.5% and 14.8%, respectively. High genetic diversity between the new species and the other species suggests it could be a new species, in comparison with previous molecular threshold (16%) used for crustacean species delimitation (Hou & Li, 2010; Lefébure *et al*., 2006).

In addition, the new species of *M. victoria* is located higher elevation at 2 654 m a.s.l. than *M. peterjaegeri* at 2 150 m a.s.l. and *M. seabri* at 1 585 m a.s.l., with up to 500 a.s.l.m elevation difference. According to their weak dispersal potential, the vertical barrier may have promoted the speciation events of the genus *Myanmarorchestia*.

Numerous differences in morphology, barcode sequences and niches give support to recognizing the new species. Accordingly, the exploration of biodiversity of Myanmar is necessary in the future.

**Key to the species of *Myanmarorchestia***

1. Coxal gills with filamentous projections, uropod Ⅱ similar in both sexes...............*M. peterjaegeri* Hou, 2017

–. Coxal gills with few filamentous projections, uropod Ⅱ sexually dimorphic...............2

2. Maxilliped palp article 2 broad............... *M. seabri* Hou, 2017

–. Maxilliped palp article 2 narrow.............. *M. victoria* **sp. nov.**

## ACKNOWLEDGEMENTS

We are grateful to Jiang-Lang Wu and Zhi-Gang Chen for their assistance in the field. We thank Prof. Hiroshi Morino, Prof. Alan Myers, Prof. Cédric d'Udekem d'Acoz, and five anonymous reviewers for their constructive comments.
